# *Bifidobacterium longum* 1714 improves sleep quality and aspects of well-being in healthy adults: a randomized, double-blind, placebo-controlled clinical trial

**DOI:** 10.1038/s41598-024-53810-w

**Published:** 2024-02-14

**Authors:** Elaine Patterson, Hern Tze Tina Tan, David Groeger, Mark Andrews, Martin Buckley, Eileen F. Murphy, John A. Groeger

**Affiliations:** 1 Novonesis, Cork, T12 N84F Ireland; 2 Nottingham Trent University, Nottingham, NG1 4FQ UK; 3 Mercy University Hospital, University College Cork, Cork, Ireland

**Keywords:** Sleep, Microbiota

## Abstract

Stress and sleep are linked with overall well-being. *Bifidobacterium longum* 1714 has been shown to influence stress responses and modulate neural responses during social stress, and influence sleep quality during examination stress in healthy adults. Here, we explored the ability of this strain to alter sleep quality in adults using subjective and objective measures. Eighty-nine adults (18–45y) with impaired sleep quality assessed with the Pittsburgh Sleep Quality Index (PSQI) and with a global score ≥ 5 were randomized to receive *B. longum* 1714 or placebo daily for eight weeks. Assessing the effect of the strain on PSQI global score was the primary objective. Secondary objectives assessed sleep quality and well-being subjectively and sleep parameters using actigraphy objectively. While PSQI global score improved in both groups, *B. longum* 1714 significantly improved the PSQI component of sleep quality (*p* < 0.05) and daytime dysfunction due to sleepiness (*p* < 0.05) after 4 weeks and social functioning (*p* < 0.05) and energy/vitality (*p* < 0.05) after 8 weeks, compared to placebo. No significant effect on actigraphy measures were observed. The 1714 strain had a mild effect on sleep, demonstrated by a faster improvement in sleep quality at week 4 compared to placebo, although overall improvements after 8 weeks were similar in both groups. *B. longum* 1714 improved social functioning and increased energy/vitality in line with previous work that showed the strain modulated neural activity which correlated with enhanced vitality/reduced mental fatigue (ClinicalTrials.gov: NCT04167475).

## Introduction

Recent work has highlighted the gut microbiota as a key contributor to healthy brain development, function, and emotional well-being throughout life^1^ possibly through the regulation of our immune, metabolic, and nervous system^2–4^. Changes in the gut microbiota composition between healthy people and those with mental health disorders such as anxiety^5–7^ and depression^8–11^ have pinpointed the gut microbiota as a potential significant target to influence mental health. More recently, the role of ‘psychobiotics’ has demonstrated considerable impact on the stress response^12–17^ and symptoms of depression^18–20^ and anxiety^21^ via the microbiota-gut-brain-axis in a strain specific manner. Such ‘psychobiotics’ may alleviate symptoms of excessive stress, anxiety, and depression by affecting physiological outputs and processes in the host such as immune function^22^, tryptophan metabolism^23^, corticosterone/cortisol^17,24^, neurotransmitters^25–27^, microbial metabolites^28^, and brain-derived neurotrophic factor (BDNF)^29^. Indeed, given the role of the gut microbiota as a key factor contributing to mood and mental health, it is reasonable to consider the gut as a window to mental well-being.

Sleep is complex and multifactorial, therefore multiple physiologic processes can influence its quality^30,31^. More recently, a growing body of evidence points to the consequences of poor sleep on immune function^32^ and the microbiota-gut-brain axis as a potential regulator of sleep health^33–35^. Adding to this evidence, changes in the gut microbiota composition have been described following sleep deprivation^36–38^ and accompanying sleep disorders^39–41^, with improvements in sleep efficiency and sleep duration contributing to enhanced gut microbiota diversity^30^. However, it should be noted that although changes in the gut microbiota composition and diversity have been described across sleep disturbances and disorders, there is a lack of uniformity regarding the compositional data across clinical studies, making it difficult today to pinpoint any specific microbial species involved in sleep health^34^. There are several routes of communication between the gut microbiome and the brain that may influence sleep such as changes in immune response, vagus nerve activation, blood–brain barrier permeability, intestinal permeability, circulating bacterial metabolite levels, and the serotonergic system (reviewed in Sen et al. 2021^35^). Whether targeting the microbiota-gut-brain axis through probiotic administration can benefit sleep health in humans is poorly understood^42^. Only a few studies to date have demonstrated placebo-controlled improvements in various aspects of sleep quality in healthy adults following consumption of specific *Lactobacillus* (sensu lato) and *Bifidobacterium* strains^16,43–47^, with other studies finding no benefit to sleep^20,48,49^. A deeper investigation into the effects of certain well-studied ‘psychobiotics’ on sleep is therefore warranted. One systematic review and meta-analysis of the effects of probiotics and heat-killed bacteria demonstrated that when sleep was assessed using what is considered the gold-standard subjective measure of sleep quality (i.e., Pittsburgh Sleep Quality Index [PSQI]), supplementation may have some efficacy in improving perceived sleep health (11 studies; n = 452)^50^. In contrast, quantified in terms of other subjective sleep measures (8 studies; n = 549) or objective measures (7 studies; n = 467), the meta-analyses did not find any reliable effect of the investigated probiotics/heat-killed bacteria^50^. Thus, whether microbial manipulation within the gut could be a reasonable target for sleep quality (both subjective and objective) in a healthy population without clinically diagnosed sleep abnormalities merits further investigation.

*Bifidobacterium longum* 1714 *(B. longum* 1714*)* has previously demonstrated positive effects on stress-, anxiety-, and depression-related behavior in pre-clinical animal studies^51,52^. In healthy adults, *B. longum* 1714 reduced perceived stress and reduced both the cortisol output and self-reported anxiety in response to acute stress^24^. This strain has also demonstrated the ability to alter brain wave activity patterns in healthy adults both in the resting state and following exposure to social stress, associated with increased energy/vitality and changes in distress, respectively^53^. In healthy students participating in semester examinations (model of prolonged stress), *B. longum* 1714 improved sleep duration (increased time spent sleeping ≥ 6 h)^54^. Considering the close relationship between stress and sleep^55^ and indeed the gut microbiota, we hypothesized that *B. longum* 1714 may improve sleep quality and metrics of well-being along the so-called stress-sleep axis. To our knowledge, this is the first placebo-controlled study to investigate the effects of a probiotic supplementation focused on both subjective and objective (actigraphy) measures of sleep quality in an adult population with impaired sleep quality. The results of the study showed that *B. longum* 1714 improved sleep quality and reduced daytime dysfunction caused by sleepiness after 4 weeks with further improvements to energy/vitality and social functioning after 8 weeks, compared to placebo.

## Materials and methods

### Study design

This was a randomized, double-blind, placebo-controlled, parallel-groups, two-arm (allocation ratio 1:1) clinical trial. Screening took place across two time-points; an online pre-screen followed by a screening visit (Visit 1 [V1]). During the online pre-screen, persons who responded to advertisements were pre-assessed for eligibility following completion of the PSQI, the Hospital Anxiety and Depression Scale (HADS), the Insomnia Severity Index (ISI), the Short Form Health Survey-36 (SF-36), and the Perceived Stress Scale (PSS). Potentially eligible persons were then invited to participate in V1 where eligibility based on inclusion and exclusion criteria was assessed (see Section “Study participants”). Demographic data and data from safety bloods, vital signs (blood pressure, heart rate, temperature), prior and concomitant medications, medical history, and anthropomorphic measurements (weight, height, body mass index [BMI]) were captured. To avoid baseline differences between groups, participants were pseudo-randomly allocated to either the *B. longum* 1714 or placebo group based on their online pre-screen scores for PSQI, SF-36, and HADS. This was done by rank ordering all persons who met the online screening criteria, first for each measure separately and then the ranks assigned to each person were averaged and ranked again on this composite measure, before final allocation to either the probiotic intervention or placebo group.

**Figure 1:**
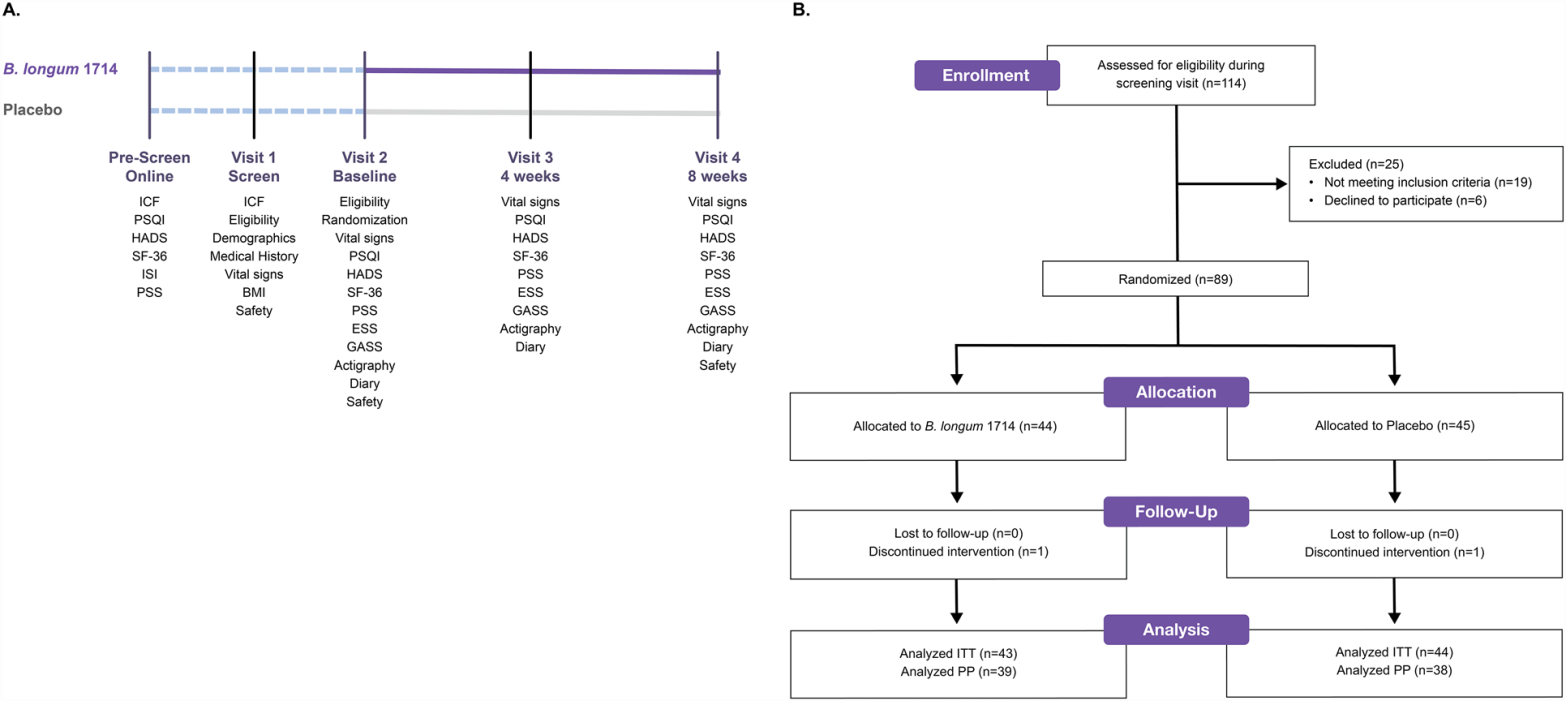
(**A**) Study design. (**B**) CONSORT flow diagram. Abbreviations: ITT, Intention-To-Treat; PP, Per-Protocol. The PP population included all the randomized participants that satisfied the inclusion/exclusion criteria, completed the study, complied with IP intake (≥ 80%), had no antibiotic use and no major protocol deviations that may have altered the treatment outcome, and who had no substantial missing data.

Once eligibility was confirmed, participants were randomized to the study at Visit 2 (V2; two weeks after V1). This was followed by an 8-week intervention with the investigational products (IP)s between V2 and Visit 4 (V4), with a 4-week time-point in between (Visit 3 [V3]). Most participants visited the study site four times during the 10-week study period, however due to travel and social distancing restrictions caused by the COVID-19 pandemic, some participants completed their V4 assessments by telephone (compliance) or online (questionnaires), with samples being collected from participants’ homes when possible. However, vital sign measures at V4 were unable to be performed outside of the study site. A detailed outline of the overall study design and investigation steps at each visit is shown in Fig. 1A.

The primary objective of the study was the change in subjective sleep quality as assessed by the PSQI global score after 8 weeks. We also investigated sleep latency, sleep duration, sleep efficiency, sleep disturbance, sleep quality, medication use, and daytime dysfunction due to sleepiness (PSQI components); waking refreshed, night-time waking, and sleep quality (daily sleep diary); daytime sleepiness (Epworth Sleepiness Scale [ESS]), and objectively assessed (actigraphy): sleep duration, sleep latency, sleep efficiency, arousals/wake episodes, and wake-time after sleep onset (WASO). Exploratory objectives included self-reported physical and mental health: overall health (SF-36), perceived stress (PSS), anxiety and depression (HADS), and a global assessment of sleep and stress (GASS). Safety objectives measured adverse events, safety blood parameters, and vital signs. The study was conducted at a single site—Atlantia Food Clinical Trials, Cork, Ireland and was first posted at ClinicalTrials.gov on 18/11/2019 under identifier NCT04167475.

### Ethics declarations

The Clinical Research Ethics Committee of the Cork Teaching Hospitals granted full approval to conduct the study on September 13, 2019. Two minor protocol amendments were also approved, with Version 2 dated February 25, 2020, and Version 3 dated March 31, 2020. The study was conducted according to the International Conference on Harmonization (ICH) Good Clinical Practice, in accordance with the most recent version of the Declaration of Helsinki on ethical principles for medical research in human subjects (ICH 1996).

### Study participants

Interested persons that underwent screening provided informed consent at each stage of the screening process (online pre-screen and V1). During the online pre-screen, participants met the following inclusion criteria: PSQI global score of ≥ 5, ISI score of < 11, HADS anxiety score of ≤ 14 and HADS depression score of ≤ 14. To be eligible for randomization at V2, all participants provided a written informed consent, were aged 18–45 years old at the time of consent with a PSQI global score of ≥ 5, an ISI score of < 11, a HADS anxiety score of ≤ 14, a HADS depression score of ≤ 14, and a BMI of ≤ 29.9 kg/m^2^. Participants must have been willing to refrain from taking any dietary supplements or other fermented foods that contained live bacteria, and any medications or preparations to improve sleep (herbal, dietary supplements, homeopathic preparations, etc.) during the study. If using products containing nicotine or caffeine, participants must have agreed to continue current usage levels and to not undertake air travel exceeding two time zones during the study. Participants must have been willing to maintain stable dietary habits and physical activity levels during the study and could communicate well with the principal investigator (PI), to understand and comply with the requirements of the study and be judged suitable for the study in the opinion of the PI. Each potentially eligible participant was evaluated by a full review of clinical history, physical examination, full blood count and routine biochemistry analysis whereby any clinically significant abnormalities in any of these tests resulted in exclusion from the study. Participants were not eligible if they had consumed antibiotics during the past 3 months, if they had a malignant- or any concomitant end-state organ disease and/or laboratory abnormalities considered by the PI to be risky or that could interfere with data collection, if they had any significant acute or chronic co-existing illness (cardiovascular, history of co-existing gastrointestinal, and/or gynecological, and/or urologic pathology [e.g., colon cancer, colitis, Crohn's disease, celiac, irritable bowel syndrome, endometriosis, prostate cancer]) or lactose intolerance. Participants were not eligible if they had any inflammatory disorders such as chronic fatigue syndrome, psoriasis, rheumatoid arthritis or any other inflammatory arthropathies, if they had a diagnosis of any psychiatric disorder other than anxiety or depression, or if they were severely immunocompromised (HIV positive, transplant participant, on anti-rejection medications, on a steroid for > 30 days, or chemotherapy or radiotherapy within the last year). Participants must not have been taking any anxiolytics, anti-depressants, anti-psychotics, anti-convulsants, centrally acting corticosteroids, opioid pain relievers, hypnotics, and/or prescribed sleep medication/herbals (e.g., valerian). Participants were deemed ineligible if they had a combined SF-36 score of >  + 2 standard deviations from the mean, if they had a history of drug and/or alcohol abuse at the time of enrolment, if they were a pregnant or lactating female, or if pregnancy as planned during the study, if they had undertaken air travel involving transit across two or more time zones in the month previous to the study, if they were shift workers or had a previous diagnosis of a sleep disorder such as sleep apnea, had any known allergy to any of the components of the IP, had a history of illicit drug use, a history of non-compliance, or had previously participated in a clinical study with an IP intervention within 60 days before screening, or had any plans to participate in another study during the study.

### Investigational products and blinding

The probiotic intervention consisted of *B. longum* 1714 at a dose of 1 × 10^9^ colony forming units (CFU), corn starch, and magnesium stearate in one capsule per day. The placebo consisted of corn starch and magnesium stearate in one capsule per day and apart from the absence of *B. longum* 1714, was otherwise identically matched to the probiotic intervention. PrecisionBiotics Group Ltd. (now part of Novonesis, Ireland) manufactured the capsules.

The study participants were instructed to take one capsule orally every morning, with or after food. An IP dispensing and return log (listing participant ID, date dispensed, amount of product, and date returned) was used to account for all IPs dispensed to and returned by the participants.

At baseline, the participants were randomized 1:1 to either the probiotic intervention or placebo group and were administered capsules containing either *B. longum* 1714 or placebo to take for the duration of the 8-week intervention. The PI, study staff at the site, and the participants were blinded to the treatment schedule. The Clinical Research Organization (Atlantia Food Clinical Trials) received a coded envelope for each participant, identifying to which arm they had been allocated. The sponsor (PrecisionBiotics Group Ltd.) also kept a set of codes for emergency use only if it became necessary to know which arm the participant had been allocated to. A broken code required the withdrawal of the participant from the study. Unblinding only occurred after all databases were locked and all statistical analyses were conducted on data that had been “locked” prior to unblinding.

### Study outcomes

#### Primary outcome

The primary objective was to evaluate the effects of 8 weeks of supplementation with *B. longum* 1714 on subjective sleep quality. The primary endpoint was the extent of change in subjective sleep quality from baseline (V2) to 8 weeks (V4), as indexed by the PSQI global score. The PSQI is a validated self-report questionnaire comprised of 19 items that measure 7 key components of sleep. Scores on each component were combined to give a global score. The PSQI global score ranges between 0 and 21, whereby a suggested cut-off global score ≤ 5 indicates good sleep quality, and a score > 5 indicates poor sleep quality^56^. Participants completed the PSQI at the online pre-screen, V2, V3, and V4.

#### Secondary outcomes: subjective assessment of sleep quality

PSQI. Sleep latency, sleep duration, sleep efficiency, sleep disturbances, sleep quality, use of sleep medication, and daytime dysfunction due to sleepiness were all individually assessed using the PSQI^56^. On these component scores, a minimum score of 0 indicated a better outcome and a maximum score of 3 indicated a worse outcome.

ESS. The ESS is a simple, self-administered questionnaire that provides a measurement of a participant’s level of daytime sleepiness^57^. There are 8 questions in the ESS whereby participants rated on a 4-point scale (0–3) their usual chances of dozing off or falling asleep while engaged in eight different activities. The ESS score (the sum of 8 item scores) ranges from 0 to 24. The higher the ESS score, the higher that person’s average daytime sleepiness. Participants completed the ESS at V2, V3, and V4.

Daily sleep diary. Participants were given access to an online diary at V2 which was completed everyday throughout the intervention period between V2 and V4. Information from the individual participants previous night’s sleep was recorded in the diary on a 5-point rating scale, including number of awakenings (“*Did you wake during the night?*”), feeling refreshed upon waking (“*How refreshed did you feel upon waking this morning?*”), and sleep quality (“*How would you rate the quality of last night’s sleep?*”). The number of diary entries were reviewed at each subsequent visit.

#### Secondary outcomes: objective assessment of sleep quality

Actigraphy is a widely used objective and continuous measure of habitual sleep and daytime activity. Participants were required to wear a wrist actimetry device (ActTrust, Condor Instruments, Brazil) throughout the intervention period between V2 and V4. Actigraphy has previously been validated against polysomnography (PSG), the gold standard objective sleep measurement^58–60^. At the end of the study, individual actigraphy files were uploaded in their machine-protected form. The complete 2 Hz datasets were downloaded from devices, and archived with their unique identifiers, dates, and times. Actimetry devices were used to measure sleep duration, sleep latency (time between lights-out and cessation of motion), sleep efficiency (total sleep time as a percentage of the sleep-period), arousal/wake episodes (numbers of blocks of contiguous wake epochs), and WASO. To identify nocturnal sleep and the endpoints specific to this study, trained personnel confirmed automatically extracted bed, sleep, and wake times. Actigraphy coders downloaded and reviewed continuous pictorial records which detailed light and activity levels, temperature, and clock time. Each file was annotated according to what time they judged that the participant prepared to go to sleep and the time they woke up. Having electronically stored both sleep and wake times, thus avoiding transcription errors, they proceeded to the next period of sleep and repeated the process. Each participant’s sleep records were scored in their entirety by two independent, blinded coders. All raw and processed data were uploaded to the data management system prior to database lock.

#### Exploratory outcomes: self-reported physical and mental health

SF-36. Participants completed the SF-36 during the online pre-screen and again at V2, V3, and V4. The questionnaire includes 36 questions to determine health and well-being from the participant’s viewpoint across 8 health domains^61^. The 8 health domains measure physical functioning, bodily pain, role limitations due to physical health problems, role limitations due to personal or emotional problems, emotional well-being, social functioning, energy/vitality, and general health perceptions^62^.

PSS. Cohen’s PSS is the most widely used psychological instrument for measuring the perception of stress^63^. The 10 questions in the PSS ask about feelings and thoughts during the last month and it measures the degree to which situations in one’s life are appraised as stressful. The total score ranges from 0 to 40, with higher scores indicating greater perceived stress. Participants completed the PSS at the online pre-screen, V2, V3, and V4.

HADS. The HADS is a self-report rating scale of 14 items on a 4-point Likert scale (range 0–3). It was designed to measure levels of anxiety (7 subscale items) and depression (7 subscale items) within the last week^64,65^. Olssøn et al. identified HADS-A ≥ 8 as an optimal cut-off for abnormally high anxiety (sensitivity 0.89, specificity 0.75), with the same cut-off for depression (sensitivity 0.80, specificity 0.88)^66^. Participants completed the HADS at the online pre-screen, V2, V3, and V4.

GASS. The Global Assessment of Stress and Sleep consisted of four single-item 11-point rating scales (ranging from 0 to 10) assessing the effects of stress and sleep on well-being over the previous week and month, whereby a higher score indicated a more severe effect (modified from Jones et al. 1996^67^). Participants completed the GASS at V2, V3, and V4.

### Safety assessment

Safety measurements included treatment-emergent adverse events (TEAEs), safety blood analyses (complete blood count and comprehensive metabolic panel), and vital signs (temperature, systolic and diastolic blood pressure, and heart rate). Safety bloods were collected at V2 and V4. Vital signs were measured at V1, V2, V3, V4, safety blood samples were collected at V1, V2, V4, and TEAEs were measured at V3 and V4. Two forms of safety analysis were performed: a statistical comparison of vital signs and safety blood analyses and scrutiny of the TEAEs reported during the study.

### Sample size calculation

A sample size of 82 allowed 80% sensitivity for detecting a treatment difference at a one-sided 0.05 significance level was calculated based on the results of Marotta et al.^44^. It was determined that 41 participants in each arm would be required to replicate the improvement in the PSQI global score following the multi-strain intervention in that study. The number of randomized participants was rounded to 90 to account for potential exclusions. The randomization allocated half of that number to each group: 45 participants to take *B. longum* 1714 each day and 45 participants to take placebo each day.

### Statistical analyses

The primary purpose of the statistical analyses was to produce robust and replicable analyses of the primary, secondary, exploratory, and safety objectives. The results were used to identify those aspects of sleep and well-being most sensitive to *B. longum* 1714 and the magnitude of this strain’s effects on subjective and objective measures of sleep. The statistical methods applied were based on the ICH document “Good Clinical Practice: Consolidated Guideline”^68^ and in accordance with the applicable Good Clinical Practice 21 Code of Federal Regulations CFR 50, 56, and 312. For variables that were measured at baseline (V2), week 4 (V3), and week 8 (V4), all analyses primarily examined differences between the *B. longum* 1714 and placebo groups in changing the dependent variable values from baseline (V2) to week 8 (V4). In addition, for the variables, the differences between placebo and *B. longum* 1714 from baseline (V2) to week 4 (V3) and week 4 (V3) to week 8 (V4) were also analyzed. These analyses were performed using a single mixed effects longitudinal analysis of the covariance model (LANCOVA). This analysis estimates the effect on the outcome variable of *B. longum* 1714 relative to the placebo at both the V3 and V4 timepoints, controlling for participants’ V2 values of the variable. For variables measured at V2 and V4 only, a standard analysis of covariance (ANCOVA) was used. This estimated the effect of *B. longum* 1714 relative to placebo on these variables at V4, controlling for V2 values of the relevant variable. Likewise, for those variables, like the sleep diary and actigraphy variables that were measured on each day from V2 to V4, their values were averaged over the days from V2 to V3 and from the days from V3 to V4. A standard ANCOVA was then used to measure the effect of *B. longum* 1714 relative to placebo on the average of the variable at the V3 to V4 stage, controlling for their values at the V2 to V3 stage. For each LANCOVA or ANCOVA analysis, estimates of the statistical model parameters (coefficients of general linear models), standard errors of these estimates, null hypothesis test statistics (t-statistics), p-values, and 95% confidence intervals were calculated. For the secondary analyses, the false discovery rate (FDR) was controlled by applying the Benjamini and Hochberg procedure^69^ at the 5% level; a maximum of 5% of the significant results will be false positives. All *p*-values in the analysis were above the calculated FDR threshold (minimum value of *p* = 0.474) and so the original p-values are included in the manuscript. However, since this was not a confirmatory trial, it was considered that the presentation of p-values without test multiplicity adjustment was sufficient. The analyses and data management were conducted using R Statistical Software (v4.0.2; R Core Team, 2019)^70^. The graphs were constructed using GraphPad Prism version 9.0 (GraphPad Software, San Diego, California, USA). The results described in the main text focus on the ITT population.

## Results

### Participants and baseline characteristics

One hundred and seventy-four (174) potentially eligible persons who met study requirements based on the online pre-screen were invited to a screening visit (V1). Of the 114 persons who attended this visit, 89 met the remaining inclusion/exclusion criteria and were subsequently randomized to the study (*B. longum* 1714; n = 44 and placebo; n = 45), which took place between January 6 and April 3, 2020 (Fig. 1B). Two participants dropped out following randomization but prior to taking any study product, therefore the statistical analysis was conducted on 87 participants: *B. longum* 1714; n = 43 and placebo; n = 44. A PSQI global score ≥ 5 taken during the online pre-screen was used to distinguish healthy people with impaired sleep quality from good sleepers, as per the inclusion criteria. Consistent with eligibility criteria during the online pre-screen, those who were randomized to placebo (mean PSQI global score = 6.4) and *B. longum* 1714 (mean PSQI global score = 6.8) were classified as having impaired sleep quality. At baseline, 56.8% of participants in the *B. longum* 1714 group and 53.3% of participants in the placebo group were below the suggested cut-off for the PSQI global score of > 5 for poor sleepers, as per the Buysse et al., 1989 cut-off scores^56^. Table 1 summarizes the baseline demographic and physiological characteristics of the 89 randomized participants. Data collected at the screening visit (V1) showed that those randomized to each group were similar on all physiological measures (Table 1). The intention-to-treat (ITT) population included all the randomized participants that satisfied the inclusion/exclusion criteria (n = 89; *B. longum* 1714, n = 44; placebo, n = 45, with n = 87 evaluable participants for the statistical analysis; *B. longum* 1714, n = 43; placebo, n = 44; Fig. 1B).

**Table 1:** Demographics and physiological characteristics at baseline.

	*B. longum* 1714		Placebo		*P*-value
	N = 44 (n = 34 Female/n = 10 Male)		N = 45 (n = 30 Female/n = 15 Male)		
	Mean	SD	Mean	SD	
Anthropometric					
Age (years)	32.43	7.91	30.13	8.41	0.19
Weight (kg)	67.21	9.62	72.00	12.35	0.04
Height (m)	1.67	0.08	1.72	0.10	0.02
BMI (kg/m^2^)	24.01	2.42	24.29	2.86	0.62
Vital signs					
Temperature (tympanic, °C)	36.35	0.40	36.18	0.37	0.04
Systolic blood pressure (mmHg)	103.75	9.01	109.71	11.41	0.01
Diastolic blood pressure (mmHg)	67.98	7.77	71.24	8.07	0.06
Heart rate (bpm)	69.39	9.26	70.02	11.20	0.77

Data included in Table 1 represent the ITT population.

*ITT* Intention-To-Treat; *N*, *n* Number of participants by treatment category (N) or sex (n); *SD* Standard Deviation.

### IP compliance and blinding

Compliance was based on the participant’s self-reported IP consumption and recorded in the electronic case report form (eCRF). The percentage of IP consumed was calculated according to the following: capsules dispensed—capsules returned*100/days since the last visit. The overall compliance in the ITT population was very high (*B. longum* 1714: 97.9%; placebo: 98.8%). Any participants whose compliance fell below 80.0% were excluded from the PP population. The randomization code remained intact for all participants for the duration of the study.

### *B. longum* 1714 did not improve the PSQI global score beyond placebo

**Figure 2:**
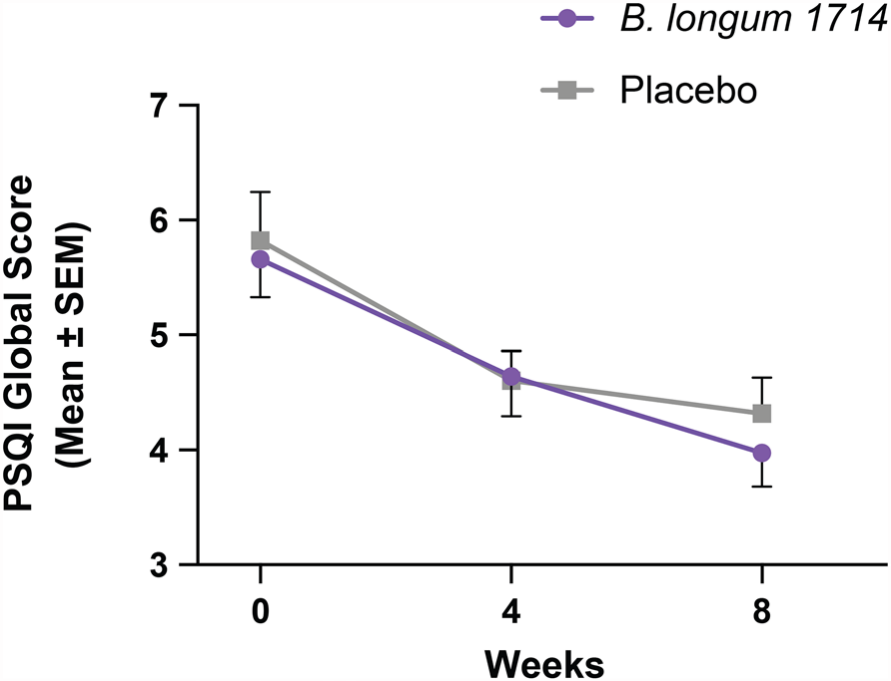
PSQI Global Score (ITT population). PSQI global score from baseline to 4 weeks and 8 weeks (Mean ± SEM). PSQI, Pittsburgh Sleep Quality Index.

Subjective sleep quality, as indicated by the PSQI global score, improved from baseline in both groups across the study (Fig. 2). *B. longum* 1714 had no effect on the change from baseline in the PSQI global score to either week 4 (*p* = 0.75) or week 8 (*p* = 0.64) compared to placebo (Table 2).

**Table 2:** Subjective measures of sleep quality—validated questionnaires.

	*B. longum* 1714		Placebo		*P*-value
	N	Mean ± SD	N	Mean ± SD	
PSQI global score					
Baseline	44	5.7 ± 2.2	45	5.8 ± 2.8	
Week 4	42	4.6 ± 2.3	43	4.6 ± 1.7	0.75
Week 8	42	4.0 ± 1.9	41	4.3 ± 2.0	0.64
Sleep quality (PSQI)					
Baseline	44	1.3 ± 0.6	45	1.2 ± 0.5	
Week 4	42	1.0 ± 0.5	43	1.2 ± 0.5	0.01
Week 8	42	0.9 ± 0.6	41	1.1 ± 0.5	0.09
Daytime dysfunction due to sleepiness (PSQI)					
Baseline	44	0.8 ± 0.6	45	0.9 ± 0.7	
Week 4	42	0.5 ± 0.6	43	0.7 ± 0.7	0.04
Week 8	42	0.4 ± 0.5	41	0.5 ± 0.7	0.25
Sleep duration (PSQI)					
Baseline	44	0.7 ± 0.7	45	0.8 ± 0.7	
Week 4	42	0.7 ± 0.7	43	0.7 ± 0.7	0.44
Week 8	42	0.6 ± 0.7	41	0.8 ± 0.8	0.63
Sleep disturbance (PSQI)					
Baseline	44	1.2 ± 0.4	45	1.2 ± 0.4	
Week 4	42	1.1 ± 0.4	43	1.0 ± 0.4	0.24
Week 8	42	1.0 ± 0.4	41	0.9 ± 0.4	0.08
Sleep medication (PSQI)					
Baseline	44	0.0 ± 0.0	45	0.0 ± 0.2	
Week 4	42	0.1 ± 0.5	43	0.0 ± 0.0	0.18
Week 8	42	0.1 ± 0.3	41	0.0 ± 0.2	0.18
Sleep latency (PSQI)					
Baseline	44	1.2 ± 0.7	45	1.2 ± 0.9	
Week 4	42	1.0 ± 0.9	43	0.8 ± 0.7	0.61
Week 8	42	0.9 ± 0.8	41	0.8 ± 0.8	0.91
Sleep efficiency (PSQI)					
Baseline	44	0.5 ± 0.8	45	0.5 ± 0.9	
Week 4	42	0.5 ± 0.7	43	0.3 ± 0.5	0.24
Week 8	42	0.3 ± 0.7	41	0.5 ± 0.8	0.36
Daytime sleepiness (ESS)					
Baseline	44	3.4 ± 2.8	45	3.7 ± 3.1	
Week 4	43	3.3 ± 3.0	44	3.5 ± 3.2	0.84
Week 8	42	3.7 ± 3.6	41	3.7 ± 3.0	0.78

ITT population. *PSQI* Pittsburgh Sleep Quality Index; *ESS* Epworth Sleepiness Scale; *SD* Standard Deviation.

### *B. longum* 1714 improved sleep quality and daytime dysfunction due to sleepiness scores after 4 weeks

**Figure 3:**
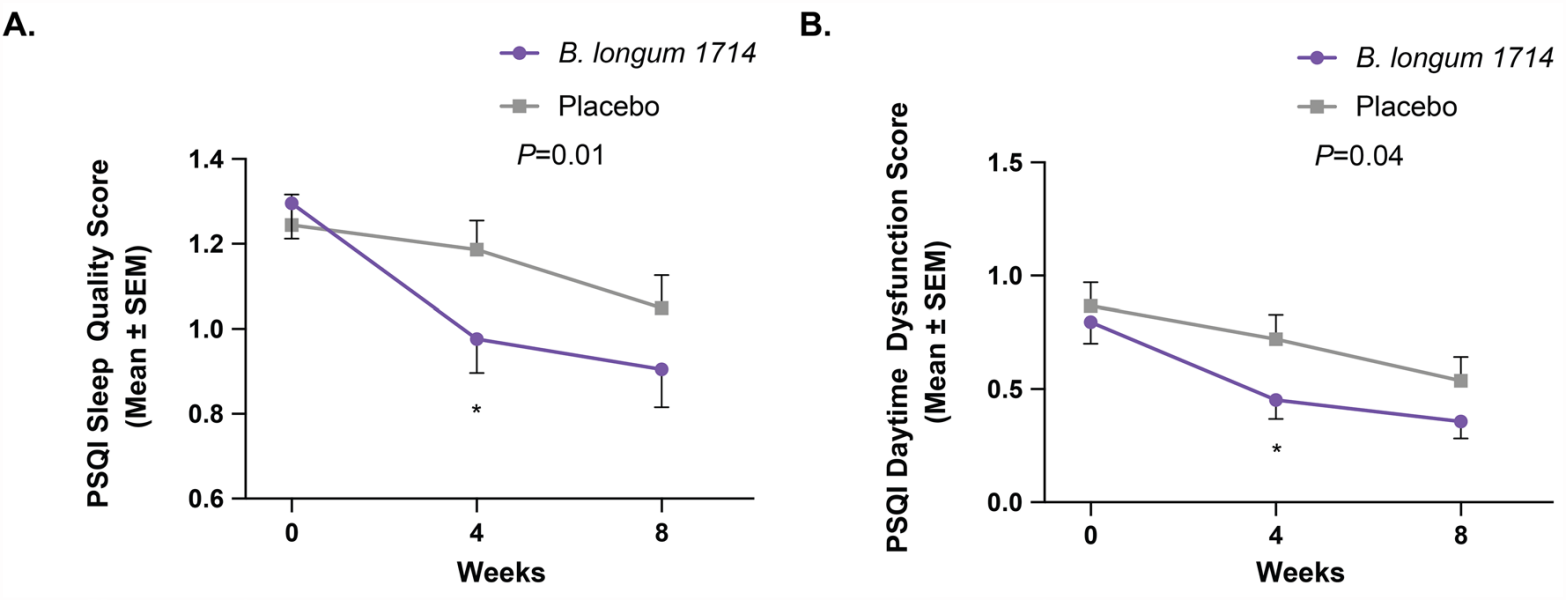
PSQI component scores (ITT population)*.* (**A**) Sleep quality score from baseline to 4 weeks and 8 weeks (Mean ± SEM). (**B**) Daytime dysfunction due to sleepiness score from baseline to 4 weeks and 8 weeks (Mean ± SEM). Statistical significance is noted in the graph. PSQI, Pittsburgh Sleep Quality Index.

The PSQI component score of sleep quality improved from baseline in both groups across the study (Fig. 3A). Participants in the *B. longum* 1714 group reported an improvement in sleep quality (decrease in PSQI sleep quality score), as indicated by a significant change from baseline to week 4, compared to the placebo group (*p* = 0.01). After 8 weeks, this result was no longer statistically significant, but trending towards (*p* = 0.09). Similarly, the PSQI component score of daytime dysfunction due to sleepiness improved from baseline in both groups across the study (Fig. 3B). Participants in the *B. longum* 1714 group reported an improvement (i.e., reduction in impairment) in daytime dysfunction due to sleepiness, as indicated by a significant change from baseline to week 4, compared to the placebo group (*p* = 0.04). After 8 weeks, this result was no longer significant (*p* = 0.25).

*B. longum* 1714 had no effect on other PSQI component scores such as sleep duration, sleep disturbance, use of sleep medication, sleep latency, or sleep efficiency after either 4 or 8 weeks compared to placebo (Table 2). Further, *B. longum* 1714 had no significant effect on daytime sleepiness as measured using the ESS after either 4 or 8 weeks compared to placebo (Table 2).

Self-reported sleep quality, waking refreshed, and the number of night awakenings were assessed using the daily sleep diary. The change from baseline to week 8 showed a trend towards an improvement in how refreshed the participants in the *B. longum* 1714 group felt throughout the study compared to those in the placebo group (Table 3; *p* = 0.07). In daily diary reports, there was no effect of *B. longum* 1714 on either the number of awakenings or on reported sleep quality throughout the study compared to placebo (Table 3).

**Table 3:** Subjective measures of sleep quality—daily sleep diary.

	*B. longum* 1714		Placebo		*P*-value
	N	Mean ± SD	N	Mean ± SD	
Waking refreshed					
Weeks 1–4	43	3.4 ± 0.5	44	3.3 ± 0.4	0.07
Weeks 5–8	43	3.5 ± 0.5	44	3.4 ± 0.4	
Number of awakenings					
Weeks 1–4	43	4.0 ± 0.4	44	3.9 ± 0.4	0.53
Weeks 5–8	43	4.0 ± 0.5	44	4.0 ± 0.4	
Sleep quality					
Weeks 1–4	43	3.6 ± 0.4	44	3.6 ± 0.4	0.15
Weeks 5–8	43	3.8 ± 0.5	44	3.6 ± 0.4	

ITT population. *SD* Standard Deviation.

### Actigraphy results demonstrated no overall effect of *B. longum* 1714 on objective measures of sleep quality at any specific timepoint

The current study also included endpoints relating to longitudinal assessments of objective sleep. The same LANCOVA modeling approach as used above was applied to actigraphy data accumulated between V2–V3 and V3–V4. Contrasts between the first and second four weeks of the study were not statistically significant (Table 4).

**Table 4:** Objective measures of sleep quality—actigraphy.

	*B. longum* 1714		Placebo		*P*-value
	N	Mean ± SD	N	Mean ± SD	
Wake episodes					
Weeks 1–4	42	16.0 ± 5.8	42	16.0 ± 5.5	0.95
Weeks 5–8	42	15.0 ± 4.6	42	16.0 ± 4.6	
Sleep efficiency					
Weeks 1–4	42	92.0 ± 4.0	42	91.0 ± 3.9	0.29
Weeks 5–8	42	92.0 ± 2.9	42	91.0 ± 2.9	
Sleep latency					
Weeks 1–4	42	83.0 ± 48.0	42	83.0 ± 47.0	0.69
Weeks 5–8	42	83.0 ± 46.0	42	88.0 ± 37.0	
Wake-time after sleep onset					
Weeks 1–4	42	0.62 ± 0.3	42	0.67 ± 0.4	0.34
Weeks 5–8	42	0.62 ± 0.2	42	0.68 ± 0.2	

ITT population. *SD* Standard Deviation.

### *B. longum* 1714 improved energy/vitality and social functioning after 8 weeks

**Figure 4:**
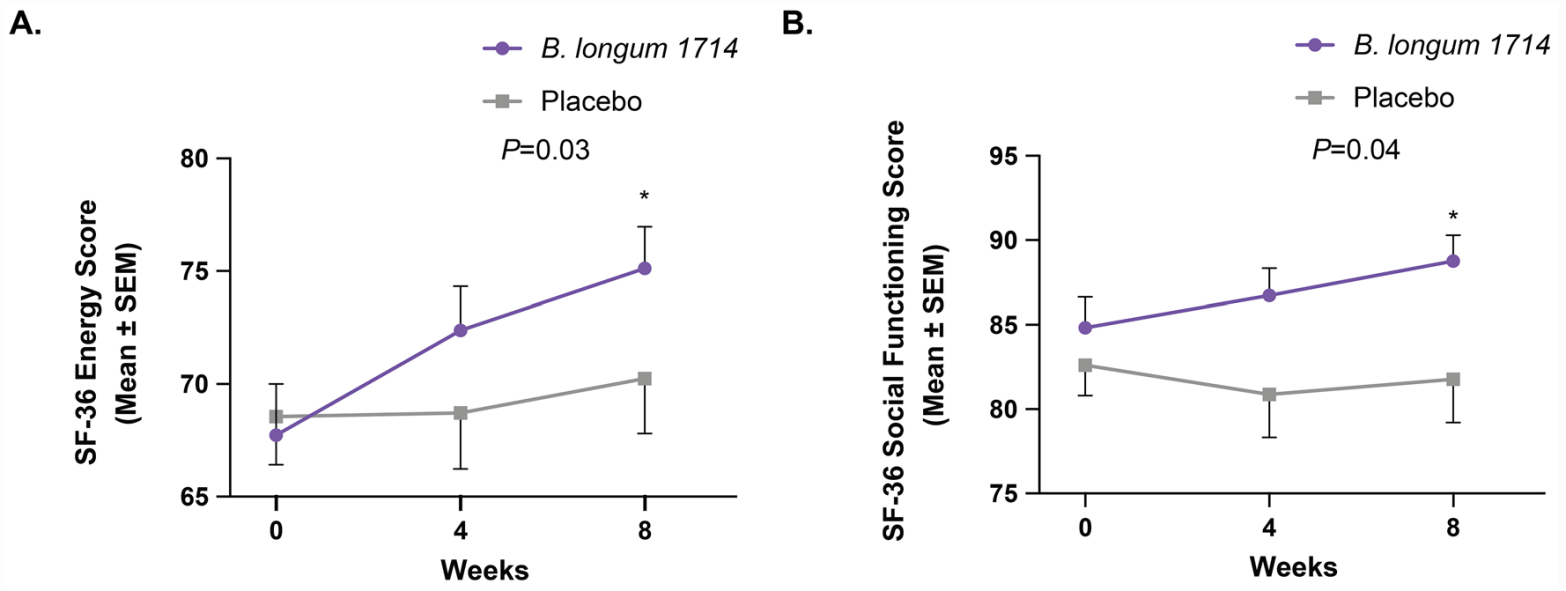
SF-36 Component Scores (ITT population). (**A**) Energy/vitality score from baseline to 4 weeks and 8 weeks (Mean ± SEM). (**B**) Social functioning score from baseline to 4 weeks and 8 weeks (Mean ± SEM). Statistical significance is noted in the graph.

The SF-36 component score of energy/vitality improved from baseline in both groups across the study (Fig. 4A). After 4 weeks, there was a trend towards a significant increase in energy/vitality in the *B. longum* 1714 group as indicated by the change from baseline to week 4, compared to the placebo group (*p* = 0.08). This trend became significant by week 8 (*p* = 0.03), indicating that participants in the *B. longum* 1714 group reported increased energy/vitality compared to those in the placebo group. Furthermore, the SF-36 component score of social functioning improved in the *B. longum* 1714 group but remained unchanged in the placebo group throughout the study (Fig. 4B). After 4 weeks, there was a trend towards a significant increase in social functioning in the *B. longum* 1714 group as indicated by the change from baseline to week 4, compared to the placebo group (*p* = 0.07). This trend became significant by week 8 (*p* = 0.04), indicating that participants in the *B. longum* 1714 group reported increased social functioning compared to those in the placebo group. Compared with baseline, there was a greater reduction (indicating greater disability) in the SF-36 component score for role limitations due to emotional problems in the placebo group compared to the *B. longum* 1714 group after 4 weeks (Table 5; *p* = 0.04). There was no effect of *B. longum* 1714 on physical functioning, role limitations due to physical health, emotional well-being, pain, or general health as measured by the SF-36 questionnaire compared to placebo (Table 5). *B. longum* 1714 had no effect on feelings of depression and anxiety as measured by HADS, or stress as measured with the PSS and GASS compared to placebo over the study (Table 6). There was however a significant reduction in the Global Assessment of Sleep (previous month score) in the *B. longum* 1714 group compared to the placebo group after 4 weeks (Table 6; *p* = 0.04) but the result was no longer significant at 8 weeks (Table 6; *p* = 0.17). This indicated that those participants in the *B. longum* 1714 group reported that lack of sleep had a lower effect on well-being after 4 weeks compared to those participants in the placebo group.

**Table 5:** Exploratory measures of physical and mental health.

	*B. longum* 1714		Placebo		*P*-value
	N	Mean ± SD	N	Mean ± SD	
Energy/vitality (SF-36)					
Baseline	44	68.0 ± 15.0	45	69.0 ± 14.0	
Week 4	42	72.0 ± 13.0	43	69.0 ± 16.0	0.08
Week 8	42	75.0 ± 12.0	41	70.0 ± 16.0	0.03
Social functioning (SF-36)					
Baseline	44	85.0 ± 12.0	45	83.0 ± 12.0	
Week 4	42	87.0 ± 10.0	43	81.0 ± 17.0	0.07
Week 8	42	89.0 ± 9.9	41	82.0 ± 16.0	0.04
Physical functioning (SF-36)					
Baseline	44	97.0 ± 5.5	45	98.0 ± 3.5	
Week 4	42	98.0 ± 5.4	43	98.0 ± 3.9	0.63
Week 8	42	98.0 ± 5.3	41	97.0 ± 10.0	0.16
Role limited through physical health (SF-36)					
Baseline	44	97.0 ± 8.0	45	97.0 ± 11.0	
Week 4	42	93.0 ± 20.0	43	89.0 ± 22.0	0.28
Week 8	42	94.0 ± 17.0	41	95.0 ± 19.0	0.78
Role limited through emotional problems (SF-36)					
Baseline	44	95.0 ± 16.0	45	97.0 ± 12.0	
Week 4	42	96.0 ± 11.0	43	88.0 ± 25.0	0.04
Week 8	42	93.0 ± 20.0	41	90.0 ± 25.0	0.32
Emotional wellbeing (SF-36)					
Baseline	44	82.0 ± 9.5	45	81.0 ± 10.0	
Week 4	42	83.0 ± 10.0	43	82.0 ± 9.7	0.48
Week 8	42	83.0 ± 11.0	41	82.0 ± 11.0	0.51
Pain (SF-36)					
Baseline	44	93.0 ± 11.0	45	90.0 ± 12.0	
Week 4	42	95.0 ± 8.2	43	90.0 ± 15.0	0.28
Week 8	42	91.0 ± 17.0	41	90.0 ± 17.0	0.93
General health (SF-36)					
Baseline	44	75.0 ± 9.0	45	75.0 ± 8.3	
Week 4	42	78.0 ± 9.5	43	75.0 ± 9.4	0.07
Week 8	42	77.0 ± 8.9	41	76.0 ± 10.0	0.36

ITT population. *SF-36* Short Form 36; *SD* Standard Deviation.

**Table 6:** Exploratory measures of mental health and well-being.

	*B. longum* 1714		Placebo		*P*-value
	N	Mean ± SD	N	Mean ± SD	
HADS: depression					
Baseline	44	1.9 ± 2.1	45	2.0 ± 1.8	
Week 4	42	1.5 ± 2.6	43	2.0 ± 1.8	0.27
Week 8	42	1.7 ± 2.5	41	1.7 ± 1.9	0.96
HADS: anxiety					
Baseline	44	4.4 ± 2.7	45	4.9 ± 2.4	
Week 4	42	3.7 ± 2.7	43	4.3 ± 2.8	0.57
Week 8	42	4.1 ± 3.6	41	4.2 ± 2.8	1.00
PSS					
Baseline	44	10.0 ± 5.6	45	10.0 ± 4.5	
Week 4	43	9.7 ± 6.0	44	11.0 ± 6.1	0.28
Week 8	42	12.0 ± 5.8	41	11.0 ± 6.3	0.85
Global assessment of stress (previous week)					
Baseline	43	2.1 ± 1.8	45	2.2 ± 1.9	
Week 4	42	2.3 ± 2.1	42	3.1 ± 2.5	0.08
Week 8	42	3.1 ± 2.4	41	2.9 ± 2.7	0.52
Global assessment of stress (previous month)					
Baseline	43	2.5 ± 1.7	45	2.4 ± 1.5	
Week 4	42	2.3 ± 1.5	42	3.0 ± 2.0	0.09
Week 8	42	2.8 ± 1.8	41	3.0 ± 2.5	0.57
Global assessment of sleep (previous week)					
Baseline	42	3.0 ± 1.8	45	2.7 ± 1.9	
Week 4	40	1.9 ± 1.8	41	2.3 ± 1.9	0.14
Week 8	40	1.9 ± 1.7	40	2.2 ± 2.5	0.26
Global assessment of sleep (previous month)					
Baseline	42	2.9 ± 1.7	45	2.6 ± 1.8	
Week 4	40	2.0 ± 1.6	41	2.7 ± 2.3	0.04
Week 8	40	1.9 ± 1.3	40	2.3 ± 2.3	0.17

ITT population. *HADS* Hospital Anxiety and Depression Scale; *PSS* Perceived Stress Scale; *SD* Standard Deviation.

### *B. longum* 1714 was safe and well-tolerated throughout the study

There was no evidence of any significant negative safety effects of *B. longum* 1714 on the study participants. One-quarter of all AEs occurred before any IP was consumed, at baseline. Seasonal conditions such as cold, cough, tonsilitis, and pneumonia represented the majority (40.0%) of cases and occurred with almost equal frequency in both groups (Supplementary Table 1). Gastrointestinal problems such as abdominal pain, gastroenteritis, and vomiting occurred in 17.0% of cases, with similar frequencies in both groups (Supplementary Table 1). Standard safety blood was collected at baseline (V2) and the end of the study (V4). The safety blood analyses revealed no major findings that would contradict the safety of *B. longum* 1714. Blood pressure, heart rate, and tympanic temperature were measured at V2, V3, and V4. No major differences were found between the groups (Supplementary Table 2).

## Discussion

The microbiota-gut-brain axis has emerged as a novel pathway that can influence sleep physiology^35^. Shifts in the composition of specific gut microbes have been identified in various sleep disorders^39–41^ and recent evidence has shown that altering the composition of the gut microbiota can improve aspects of sleep quality^44,54,71,72^. Manipulation of the gut microbiota through microbial intervention is, therefore, a novel approach to influence sleep and well-being. *B. longum* 1714 was selected as the candidate probiotic intervention for this study based on previous data reporting its positive effect on stress and potentially sleep. The results of this study demonstrated that *B. longum* 1714 could improve sleep quality and reduce daytime dysfunction caused by poor sleep after 4 weeks, while also increasing energy/vitality and improving social functioning after 8 weeks, compared to placebo.

The primary objective was to evaluate the effect of 8 weeks supplementation with *B. longum* 1714 on subjective sleep quality (PSQI global score) in healthy adults with impaired sleep quality. The PSQI global score has previously been used in other studies to determine how microbiome modification can affect sleep^54,73–75^. A recent systematic review and meta-analysis also described the PSQI global score as the gold-standard subjective measure of sleep quality which could detect the effects of probiotic and parabiotic supplementation on sleep^50^. In this study, the PSQI global score improved from baseline in both groups, but *B. longum* 1714 had no superior effect over that of placebo. This result is in line with other studies that were unsuccessful in detecting a placebo-controlled improvement in the PSQI global score following various probiotic interventions, including *B. longum* 1714^44,54,75^. Interestingly, a transient effect on sleep quality was previously demonstrated in an open label study in females with irritable bowel syndrome (IBS), where *B. longum* 1714 in combination with *B. longum* 35624 improved the PSQI global score after 4 weeks without a further improvement at week 8^15^. Of note, 31.8% of participants in the *B. longum* 1714 group and 37.8% of participants in the placebo group improved their PSQI global scores between the online pre-screen and baseline and were randomized to the study with PSQI global scores of ˂ 5. The improvement of PSQI global scores between visits was surprising and we hypothesize that it may be due to PSQI global score being a labile subjective measure. It may also be that healthy people with impaired sleep quality but not sufficiently bad to achieve a score of 11 on ISI are especially labile. Despite the average scores meeting the inclusion criteria for each group, it is worth highlighting that a significant proportion of the population were outside the inclusion criteria when randomized to the study. Furthermore, while the target population in our study was healthy adults with impaired sleep quality (defined in this study as a PSQI global score ≥ 5), the scores of a substantial percentage of participants in both groups were below the suggested cut-off for the PSQI global score of > 5 for poor sleepers at baseline. Therefore, a significant proportion of the population included in the study were good sleepers as per the suggested cut-off described by Buysse et al. 1989^56^, thus limiting the scope for demonstrating a placebo-controlled improvement. Furthermore, the extent of the placebo effect may have been underestimated with a large increase observed particularly from week 4 to week 8. Interestingly, it was previously demonstrated that assigning a state of sleep quality, e.g., “below average” or “above average” based on time spent in rapid eye movement (REM) sleep to participants’ rather than the participants’ actual self-reported sleep quality could influence cognitive performance, demonstrating that placebo effects can be driven by an individual’s mindset and that self-reported sleep quality is a component of well-being that may be vulnerable to placebo effects^76^.

A significant improvement in both sleep quality and daytime dysfunction due to sleepiness (PSQI component scores) after 4 weeks intervention with *B. longum* 1714 compared to placebo was observed. Participants were asked to rate their overall sleep quality and to report the frequency by which they had difficulty staying awake while performing tasks/activities such as driving, engaging in social activity, and maintaining enthusiasm to get things done within the last month. This result is particularly interesting because poor sleep quality and excessive daytime sleepiness co-exist and can have profound effects on cognitive function. It has been shown that excessive daytime sleepiness caused by poor sleep quality can negatively affect behavior, job performance and academic achievement^77–79^. Furthermore, it was previously shown in mice that sleep disruption had an impact on cognitive function which could be restored following probiotic intervention^80^, thus suggesting a role of the microbiota-gut-brain axis. Despite the challenge of placebo effects in this study, *B. longum* 1714 improved both sleep quality and daytime dysfunction throughout the study, and reached statistical significance compared to placebo as early as after 4 weeks intervention. This is in line with previous clinical findings in models of stress that detected an effect of the strain at 4 weeks^24,53^. Thus, while *B. longum* 1714 consumption did not significantly improve overall sleep quality as measured with the PSQI global score compared to placebo, this strain has demonstrated significant placebo-controlled efficacy, improving individual components of the PSQI such as sleep duration^54^ and both sleep quality and daytime dysfunction due to sleepiness in this study. This raises the question as to whether individual components of the PSQI may be more sensitive for detecting probiotic effects on individual aspects of sleep rather than with a global score.

*B. longum* 1714 supplementation improved energy/vitality levels, reaching significance compared to placebo after 8 weeks. Furthermore, *B. longum* 1714 supplementation improved social functioning after 8 weeks, meaning that those participants in the *B. longum* 1714 group reported less limitations to social activities caused by emotional or physical problems. The effect of *B. longum* 1714 on energy/vitality was previously reported, whereby significant changes in brain wave activity patterns in specific brain regions in the resting state correlated with increased energy levels after 4 weeks—a result not detected in the placebo group^53^. The current study corroborates our previous finding on the role of *B. longum* 1714 on energy/vitality and identifies this strain as a probiotic that supports overall mental well-being. To our knowledge, this result has not been demonstrated for any other probiotic strain, and therefore differentiates *B. longum* 1714 from other studied strains which have not shown any effect on energy/vitality in subjects with IBS, subjects with fibromyalgia, or the elderly^45,81,82^. In fact, only lifestyle changes such as 16 weeks of aerobic exercise regimes or use of hypnotics such as eszopiclone have previously demonstrated similar improvements over the previous 4 weeks in sleep quality, daytime dysfunction, and feelings of “pep” and energy all the time^83,84^. Whether the effect of *B. longum* 1714 on energy/vitality was linked to improved outcomes of sleep quality and daytime dysfunction is not clear but worthy of consideration for a delayed benefit in well-being.

A limitation of this study was the absence of a positive effect of *B. longum* 1714 on the objective measures of sleep quality measured using actigraphy. Of note, objective and subjective data for sleep quality frequently do not correlate in the literature. In an open-label pilot study, a positive effect of the probiotic combination of *L. helveticus* R0052 and *B. longum* R0175 on subjective sleep quality was not similarly reported for objective sleep quality^85^. Furthermore, the systematic review and meta-analysis from Irwin and colleagues found that probiotics may be efficacious in improving perceived/subjective sleep quality, measured via PSQI, but not objective sleep quality, measured via actigraphy^50^. Although we found no effect of *B. longum* 1714 on objective measures of sleep quality in this study, previous results have shown that *B. longum* 1714 could be effective at the level of the brain by significantly increasing slow brain wave activity in both the resting state (theta wave) and in response to social stress (theta and alpha wave) in specific brain regions in healthy adults^53^. Finally, the significance of the secondary outcomes should be treated as exploratory that merit further analysis in future studies as FDR multiplicity correction should be considered.

The effects of *B. longum* 1714 on stress have been well-documented. We previously demonstrated that this strain significantly reduced both the psychological and physiological response to acute stress and reduced perceived stress during a 4-week intervention^24^. *B. longum* 1714 also altered brain wave activity patterns in healthy adults experiencing social stress, which may influence brain function during stressful times^53^. Stress affects sleep^86^ and vice versa^87^ and anxiety can lead to poor sleep quality, but in the current study, whether reduced stress may have resulted in improved sleep quality warrants further investigation since there was also no observed effect of the intervention on stress or mood. The challenge in observing a reduction in stress or improvement in mood following probiotic intervention in healthy populations has previously been demonstrated^48,54^ and this study supports this challenge. At baseline, the participants in both groups demonstrated low stress (PSS scores) and mood was considered good/normal (HADS-depression scores). In addition, the current study began in early January 2020 and ended in early April of the same year. Importantly, the COVID-19 pandemic may have been a confounder on the stress endpoints. Future interventional studies with *B. longum* 1714 should therefore focus on investigating the effect of this strain in populations with less rested lifestyles and mild/early symptoms of excess stress, anxiety, low mood, and/or unhealthy sleep.

The mechanisms through which probiotics and other gut microbiome modulators may influence sleep health via the microbiome-gut-brain axis have recently been described^35^. Microbial metabolites within the gut, the serotonergic system, the vagus nerve, and peripheral immune reactions can all regulate sleep via the microbiome-gut-brain axis^35^. Furthermore, the relationship between stress, sleep, and the immune system is well established. The hypothalamic pituitary adrenal (HPA) axis, autonomic nervous system, and the enteric nervous system all directly interact with the immune system^13,22^. Stress is associated with increased inflammation and as such, chronic low-grade inflammation is a biological driver of adverse health outcomes associated with stress^88^. Stress can also have an impact on the environment of the gut and the composition of the gut microbiota, while probiotic administration can influence the stress response^13^. Sleep is also considered an important modulator of the immune system, whereby disrupted sleep is associated with a pro-inflammatory state^89,90^. Viral and bacterial infections can also have a negative impact on sleep health^91^. Taken together, this suggests a bidirectional route of communication via the microbiome-gut-brain axis, which supports the close relationship between stress, sleep, and immunity. The immunoregulatory effects of probiotics have been proposed to occur through the generation of T regulatory cell populations and the synthesis and secretion of the anti-inflammatory cytokine, IL-10^12,92^. In a recent study in females with IBS, *B. longum* 1714 in combination with *B. longum* 35624 improved anxiety, depression, and sleep quality which was associated with a decrease in the pro-inflammatory cytokine TNF-α^15^. Thus, it is plausible that the positive effects of *B. longum* 1714 on stress and sleep occur through immunoregulation within the peripheral immune system and that these signals are communicated to the brain either directly or indirectly via the microbiome-gut-brain axis. Furthermore, the microbial production of tryptophan which can in turn increase serotonin availability in both the peripheral and central nervous system^93^ can promote healthy sleep^93–95^, as serotonin can be converted to melatonin, the key molecule with hormonal properties that regulates sleep/wake cycles^96^. It was previously shown that 6 weeks administration of a probiotic mixture containing *B. longum* increased salivary melatonin levels^97^. Considering the proposed mechanisms through which *B. longum* 1714 can have a positive effect on stress, sleep, and energy, future studies investigating the effect of this strain on overall mental wellness should focus on underpinning the specific mechanism of action to determine whether our hypotheses can be supported.

Overall, this study has demonstrated that the *B. longum* 1714 strain, which has previously demonstrated promising effects on stress, may also benefit well-being and sleep. *B. longum* 1714 was well tolerated and improved both sleep quality and daytime dysfunction after 4 weeks and improved energy/vitality and social functioning after 8 weeks. Considering both sleep and stress are highly placebo-responsive, these results in a healthy population are highly encouraging and call for additional investigation to better understand the capacity of *B. longum* 1714 to improve sleep and other mental wellness-related health indications. The development of novel interventions to improve sleep health, and particularly probiotics that positively affect the gut microbiome without negatively impacting other functions, will contribute to the overall well-being of individuals, because sleep is a fundamental measure of health.

### Supplementary Information

Supplementary Tables.

## Data Availability

The datasets generated during and/or analyzed during the current study are available from the corresponding author upon reasonable request.
