# Supplementary material for: Bifidobacterium longum 1714 improves sleep quality and aspects of well-being in healthy adults: a randomized, double-blind, placebo-controlled clinical trial
Source: Sci Rep. 2024 Feb 14;14:3725. doi: 10.1038/s41598-024-53810-w (PMC10866977; doi:10.1038/s41598-024-53810-w)
Supplement: Supplementary file 1 — Supplementary Tables. [file 41598_2024_53810_MOESM1_ESM.docx]

***Bifidobacterium longum* 1714 improves sleep quality and aspects of well-being in healthy adults: a randomized, double-blind, placebo-controlled clinical trial.**

**Elaine Patterson^1*^, Hern Tze Tina Tan^1*^, David Groeger^1^, Mark Andrews^2^, Martin Buckley^3^, Eileen F. Murphy^1#^, John A. Groeger^2^**

^1.^ Novonesis, Cork, T12 N84F, Ireland

^2.^ Nottingham Trent University, Nottingham NG1 4FQ, United Kingdom

^3.^ Mercy University Hospital, University College Cork, Cork, Ireland

* These authors contributed equally to this work.

^#^ Correspondence and requests for materials should be addressed to: eilm@novozymes.com

**Included:**

Supplementary Table 1. Adverse events

Supplementary Table 2. Vital signs

**Supplementary Table 1. Adverse events**

­

|  | ***B. longum* 1714**  **(N=14)** | | | **­Placebo**  **(N=15)** | | |
| --- | --- | --- | --- | --- | --- | --- |
|  | **Baseline** | **Week 4** | **Week 8** | **Baseline** | **Week 4** | **Week 8** |
| Common cold | 3 | 3 | 0 | 1 | 5 | 1 |
| Fainting | 0 | 0 | 0 | 2 | 0 | 0 |
| Pneumonia | 1 | 0 | 0 | 0 | 0 | 0 |
| Cough | 0 | 0 | 0 | 1 | 0 | 0 |
| Tonsillitis | 0 | 0 | 0 | 0 | 1 | 0 |
| Abdominal pain | 0 | 0 | 0 | 0 | 0 | 1 |
| Gastroenteritis | 0 | 0 | 1 | 0 | 0 | 0 |
| Vomiting | 0 | 2 | 0 | 0 | 2 | 1 |
| Headache | 1 | 0 | 1 | 0 | 1 | 0 |
| Migraine | 0 | 0 | 1 | 0 | 0 | 1 |
| Tachycardia | 0 | 1 | 0 | 0 | 0 | 0 |
| Menstruation irregular | 0 | 1 | 0 | 0 | 0 | 0 |
| Absence of menstruation | 0 | 1 | 0 | 0 | 0 | 0 |
| Intervertebral disc bulging | 0 | 0 | 0 | 0 | 0 | 1 |
| Carpal tunnel syndrome | 0 | 0 | 1 | 0 | 0 | 0 |
| Hand fracture | 0 | 0 | 0 | 0 | 0 | 0 |
| Human bite | 0 | 0 | 0 | 0 | 1 | 0 |
| TOTAL | 5 | 8 | 4 | 4 | 10 | 5 |

Adverse events, ITT population.

**Supplementary Table 2. Vital signs**

|  | ***B. longum*1714** | | **Placebo** | |  |
| --- | --- | --- | --- | --- | --- |
|  | **N** | **Mean ± SD** | **N** | **Mean ± SD** | ***P*-value** |
| **DIASTOLIC BLOOD PRESSURE (mmHg)** | | | | | |
| Baseline | 44 | 69.0 ± 8.1 | 45 | 69.0 ± 9.3 |  |
| Week 4 | 43 | 70.0 ± 7.6 | 44 | 70.0 ± 8.8 | 0.89 |
| Week 8 | 26 | 70.0 ± 7.9 | 35 | 69.0 ± 7.6 | 0.45 |
| **SYSTOLIC BLOOD PRESSURE (mmHg)** | | | | | |
| Baseline | 44 | 106.0 ± 9.3 | 45 | 107.0 ± 11.0 |  |
| Week 4 | 43 | 106.0 ± 9.4 | 44 | 109.0 ± 8.8 | 0.29 |
| Week 8 | 26 | 107.0 ± 12.0 | 35 | 107.0 ± 9.1 | 0.97 |
| **HEART RATE (bpm)** | | | | | |
| Baseline | 44 | 69.0 ± 12.0 | 45 | 70.0 ± 11.0 |  |
| Week 4 | 43 | 70.0 ± 12.0 | 44 | 70.0 ± 11.0 | 0.85 |
| Week 8 | 26 | 70.0 ± 9.6 | 35 | 68.0 ± 11.0 | 0.42 |
| **TEMPERATURE (TYMPANIC; °C)** | | | | | |
| Baseline | 44 | 36.0 ± 0.4 | 45 | 36.0 ± 0.3 |  |
| Week 4 | 43 | 36.0 ± 0.5 | 44 | 36.0 ± 0.4 | 0.04 |
| Week 8 | 26 | 36.0 ± 0.5 | 35 | 36.0 ± 0.5 | 0.50 |

Vital signs, ITT population. Abbreviations: SD, Standard Deviation
